# Ultrasound-guided localization of the radial nerve along the humerus: providing reference points for safer upper arm surgery

**DOI:** 10.1007/s12306-024-00841-1

**Published:** 2024-07-23

**Authors:** T. Da Silva, D. Mueck, C. Knop, T. Merkle

**Affiliations:** 1 University of Tuebingen, Tuebingen, Germany; 2 Klinikum Stuttgart, Stuttgart, Germany

**Keywords:** Sonographic identification, Radial nerve, Upper arm anatomy, Ultrasound, Iatrogenic radial nerve palsy, Humerus shaft fractures

## Abstract

**Purpose:**

The close proximity of the radial nerve to the humerus poses a risk during upper arm surgery. Although the general course of the radial nerve is well-known, its exact position in relation to anatomical reference points remains poorly investigated. This study aimed to develop a standardized protocol for the sonographic and clinical identification of the radial nerve in the upper arm. The ultimate goal is to assist surgeons in avoiding iatrogenic radial nerve palsy.

**Methods:**

A total of 76 measurements were performed in 38 volunteers (both sides). Ultrasound measurements were performed using a linear transducer (10 MHz) to identify the radial nerve at two key points: RD (where the radial nerve crosses the dorsal surface of the humerus) and RL (where the radial nerve crosses the lateral aspect of the humerus). Distances from specific reference points (acromion, lateral epicondyle, medial epicondyle, olecranon fossa) to RD and RL were measured, and the angle between the course of the nerve and the humeral axis was recorded. Humeral length was defined as the distance between the posterodorsal corner of the acromion and the lateral epicondyle.

**Results:**

The distance from the lateral epicondyle to RD was on average 15.5 cm ± 1.3, corresponding to 50% of the humeral length. The distance from the lateral epicondyle to RL was on average 6.7 cm ± 0.8, corresponding to 21% of the humeral length. The course of the nerve between RD and RL showed an average angulation of 37° to the anatomical axis of the humerus. Gender, BMI, dominant hand, and arm thickness did not correlate with the distances to RD or RL. Measurements were consistent between the left and right side.

**Conclusion:**

The radial nerve can typically be identified by employing a 1/2 and 1/5 ratio on the dorsal and lateral aspects of the humerus. Due to slight variations in individual anatomy, the utilization of ultrasound-assisted visualization presents a valuable and straightforward approach to mitigate the risk of iatrogenic radial nerve palsy during upper arm surgery. This study introduces an easy and fast protocol for this purpose.

## Introduction

The close location of the radial nerve to the humerus exposes this nerve to a special risk both for humerus fractures and during its surgical treatment. The iatrogenic rate of a radial lesion is reported at 6% after humeral proximal shaft fractures treated with a lateral plate osteosynthesis, 14% after treatment of mid-shaft fractures with a dorsal plate and 10% after temporary fixation with external fixation of distal humerus fractures [1–3]. Deep knowledge of the anatomy of the radial nerve is therefore essential for upper arm surgery. In general, the course of the radial nerve is known and can be described as followed: the radial nerve has its origin in the posterior fasciculus of the brachial plexus; in its further course it winds helically around the dorsal part of the humerus in the sulcus radialis accompanied by the profunda brachii artery; it then passes through the septum intermuscular, between the brachioradialis muscle and brachialis muscle in the so-called radial tunnel into the elbow, where it divides into two parts (ramus superficialis and ramus profundus.

Despite the overall understanding of the radial nerve’s general course, there is limited research on its specific position relative to important surgical landmarks. Of particular importance for humerus shaft fracture surgeries are the locations of the radial nerve on the dorsal and lateral surfaces of the humerus. The identification, preparation, and protection of the nerve are critical in the dorsal approach for plate osteosynthesis, while its presence at the lateral side can pose challenges with an extended deltopectoral approach for long plates or percutaneous Kirschner wire fixation for pediatric distal humerus fractures, among other procedures [1].

Although a few cadaver-based studies have described these two positions [4–8], several unresolved obstacles exist in their implementation. Firstly, there is a lack of standardized reference points for measuring the distance to the radial nerve. Previous studies have utilized various reference points such as the medial or lateral epicondyle, humerus head, acromion, or olecranon fossa. Secondly, the commonly used absolute distance measurement in centimeters may not provide optimal clinical value. Instead, a relative value expressed as a percentage of the humeral length would be more clinically relevant [9]. Thirdly, in the clinical setting, the normal anatomy is often disrupted by factors like swelling, fractures, tumors, or patient cooperation issues [4]. Furthermore, there is inherent uncertainty in extrapolating cadaver-based measurements to patients in the clinical setting due to their specific anatomical variations, particularly in the distal upper arm [5].

To address these challenges, an ultrasound-based method can be employed to accurately identify the course of the radial nerve. The objective of this study is to establish a standardized protocol for the reliable sonographic identification of the radial nerve. Additionally, we aim to describe its course in a clinically-oriented manner that aids surgeons in predicting the nerve’s location.

Our hypothesis suggests that by integrating clinical reference points and standardized ultrasound visualization, the identification of the radial nerve along the dorsal and lateral aspects of the humerus can be easily approached.

## Methods

To collect detailed information on the location of the radial nerve along the humerus, a standardized approach was used to identify the nerve with ultrasound and measure its distances to key reference points that are clinically easy to find (such as the medial and lateral epicondyle, acromion, acromioclavicular joint and the olecranon fossa). Measurements were performed in the prone position, being this is the most common position for humerus shaft and distal humerus surgery. The focus of the measurements were the location of the radial nerve on the dorsal and lateral aspect of the humerus, since these are the most relevant for upper arm surgery. A minimum of 70 measurements were calculated to ensure a statistical power of 90%. The selection of the population was done on a volunteer basis, and no financial or other rewards were provided. Informed consent was obtained from all participants. Inclusion criteria consisted of volunteers aged 18 years and older with no previous upper arm surgery, while exclusion criteria included current or prior infections, tumors, or fractures in the measured upper arm. Both sides were examined, and patient data was pseudonymized. A single examiner performed the measurements using a 10 MHz linear transducer on a Siemens ACUSON X 300 ultrasonic device.

### Measurement protocol

#### Marking the bone reference points (seated position)

Initially, reference points are identified and marked on both arms of the seated subject using a water-soluble skin marker. These points include the acromioclavicular joint (AC joint), the posterolateral edge of the acromion (Acr.), the lateral epicondyle (LE), the medial epicondyle (ME), and the olecranon fossa (OF). The distance between the posterolateral edge of the acromion and the lateral epicondyle serves as an estimate of the humeral length (HL). Ultrasound is employed to locate the olecranon fossa, which is identified as the point where the distal dorsal convexity of the humerus transitions into the characteristic concavity of the fossa.

#### Identifying the radial nerve (prone position)

**Fig. 1:**
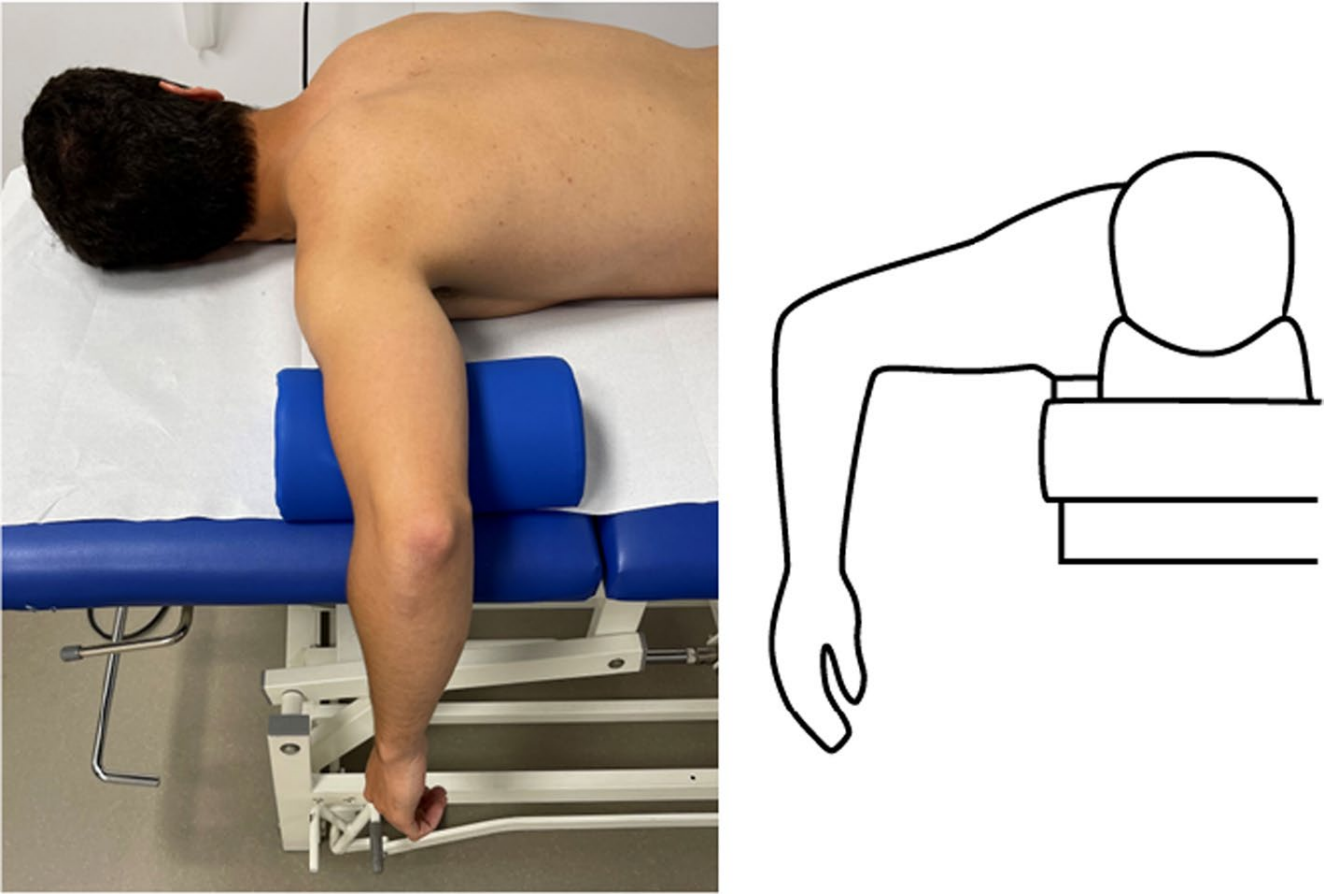
Volunteer in prone position with the left shoulder in 90° abduction and elbow in 90° flexion

The subject is placed face down on an examination table in the prone position. The examined shoulder is abducted at 90° and the elbow flexed at 90° (Fig. 1).

**Fig. 2:**
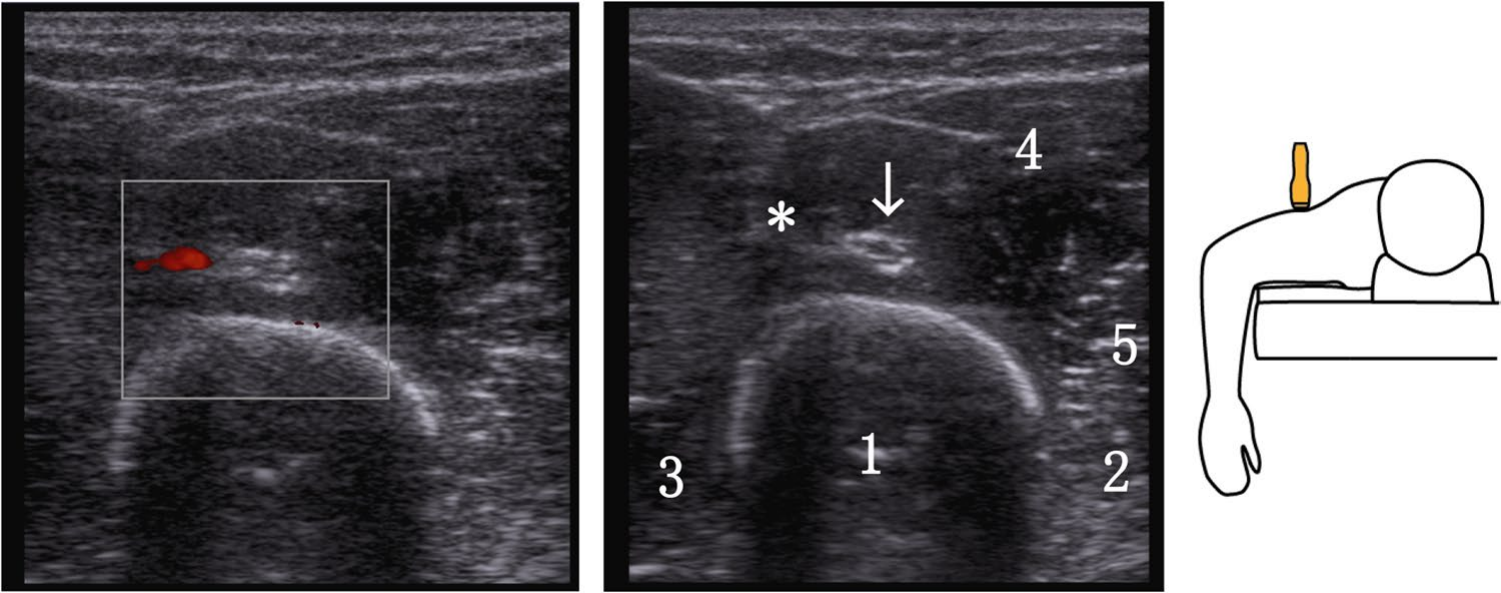
Left: sonography with Doppler showing the radial nerve seen on the dorsal aspect of the humerus (RD) accompanied by the deep brachial artery. Middle: same image without Doppler. Legend: arrow radial nerve, * deep brachial artery, 1 humerus, 2 brachial muscle, 3 triceps brachii muscle (medial head), 4 triceps brachii (medial head), 5 lateral intermuscular septum. Right: schematic representation of the position of the transducer for the dorsal examination

*Dorsal aspect of the humerus *(*RD*) Using a linear transducer, the examiner identifies the crossing point of the radial nerve with the dorsal surface of the humerus and marks it on the skin using a water-soluble skin marker. To facilitate nerve identification, begin scanning at the proximal 1/3 of the humerus’s dorsal side in the sagittal plane, gradually moving distally. Typically, the nerve runs alongside the deep brachial artery (a branch of the brachial artery), which serves as a reliable guide. Distinguishing the radial nerve from adjacent vessels can be aided by employing color-coded Doppler sonography (CCDS). The marked RD point corresponds to the site where the radial nerve crosses the dorsal aspect of the humerus, typically positioned at approximately half the length of the humerus (Fig. 2).

**Fig. 3:**
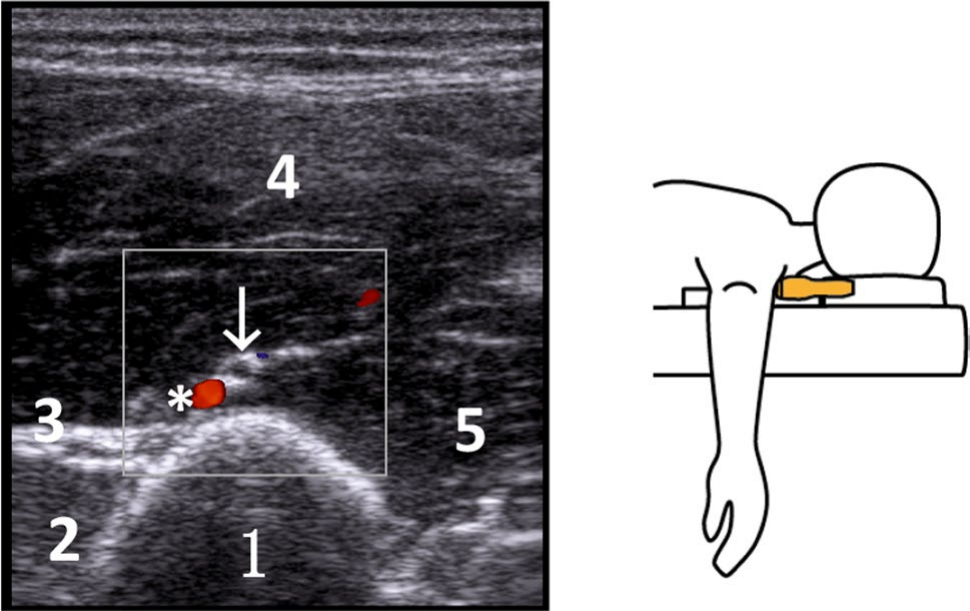
Left: sonography with Doppler of the radial nerve (arrow), accompanied with a radial collateral artery (*) visualized in the anterior compartment at the lateral side of the humerus. Legend: 1 humerus, 2 biceps brachii muscle, 3 lateral intermuscular septum, 4 brachial muscle, 5 triceps brachii muscle. Right: schematic representation of the position of the transducer for the lateral examination

*Distal*-*lateral aspect of the humerus *(*RL*) The nerve is traced distally as it moves away from the humerus in a lateral direction and traverses the lateral intermuscular septum into the anterior compartment, where it is enveloped by the brachial muscle. The crossing of the radial nerve over the lateral aspect of the humerus (RL) generally occurs around the distal 1/5 of the humeral length. At this juncture, the deep brachial artery may remain visible, or it may have already bifurcated into its radial collateral branches (Fig. 3).

#### Measuring the distances

**Fig. 4:**
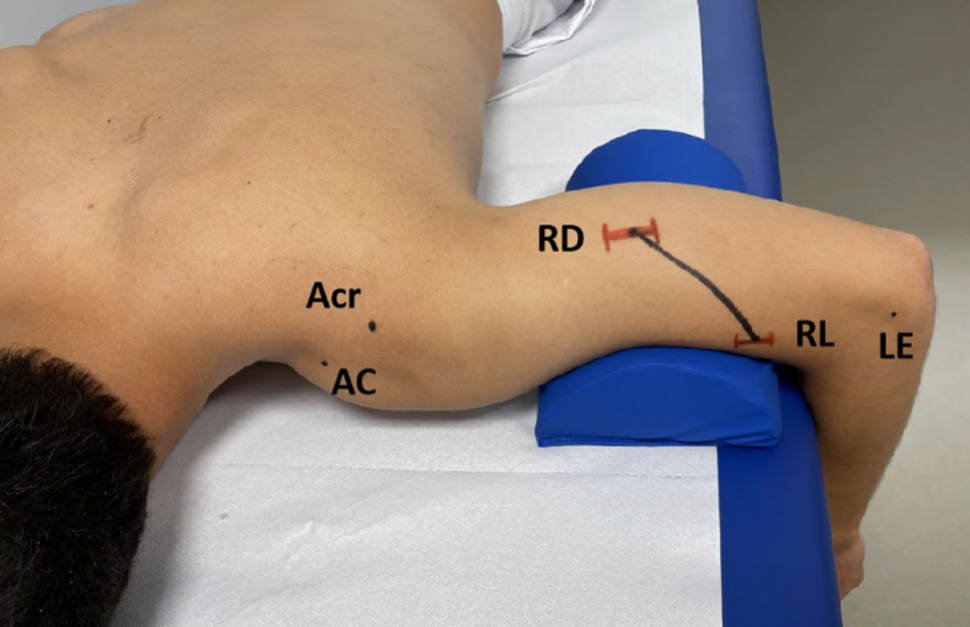
Example of the typical landmarks on the left upper arm of a volunteer in prone position. In this case, the dorsal and lateral position of the radial nerve coincided with the average distance to the main landmark (around 50% and 21% to EL). The standard deviation was drawn in red for educational purposes (RD-EL 1.3 cm and RL-EL 0.8 cm)

**Fig. 5:**
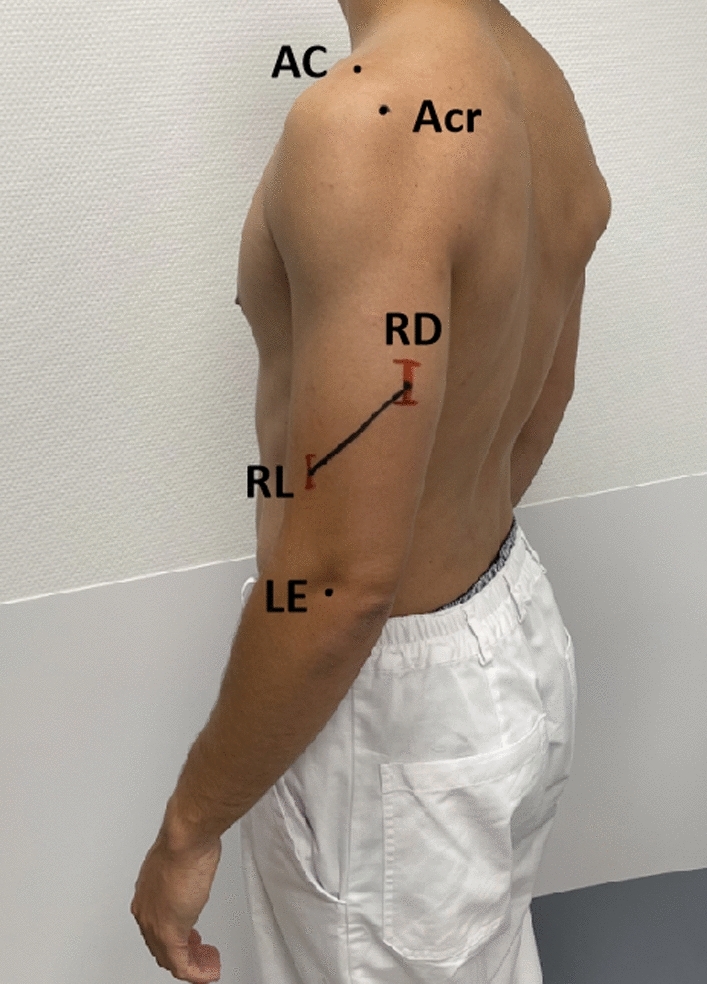
Same volunteer seen from a different perspective, in standing position, for illustrative purposes

The distances between the landmarks (ME, LE, OF, and Acr.) and the two measurement points of the radial nerve (RD and RL) are assessed using a flexible measuring tape. A straight line is drawn to connect the two points RD and RL (Figs. 4, 5). To determine the angle between the nerve’s trajectory and the humeral axis, an orthopedic protractor is utilized.

### Statistical analysis

Statistical analysis was performed using JASP (0.11.1/October 7, 2019–jasp.stats.org) provided by the University of Amsterdam (Amsterdam, The Netherlands). The significance level was set at *p*-value = 0.04.

## Results

Overall, 76 measurements were made (38 subjects, with both sides examined). This included adults of all age spectrums (from 20 to 90). As expected, humeral length varied according to general height. Arm thickness/circumference was measured at 5, 10 and 15 cm proximally from the EL and did not correlate to the position of the radial nerve at RD or RL. The distance from the lateral epicondyle (LE) until the crossing of the radial nerve on the dorsal side of the humerus (RD) was on average 15.5 cm ± 1.3, which corresponded to 50% of the humeral length. The distance from the LE until the crossing of the radial nerve on the lateral side of the humerus (RL) was on average 6.7 cm ± 0.8 cm, corresponding to 21% of the humeral length. The course of the nerve between RD and RL showed an average angulation of 37° to the anatomical axis of the humerus.

Gender, BMI, dominant hand or arm thickness did not correlate to the distances to RD or RL (*p-*value > 0.04). Moreover, it is relevant to state that the distances to RD and RL were not different among sides within the same individual (*p-*value > 0.04). A summary of the measurements can be shown in Tables 1 and 2.

**Table 1:** Summary of study population characteristics

	Total (76)	Mean	[min–max] ± SD
Gender (female)	68%	–	
Age (year)		35.8	[20–90] ± 19
Dominant hand (right)	89%		
Side (right)	50%		
BMI		23	[17–34] ± 3.3
Humeral length (cm)		31.1	[27.5–36.3.3] ± 1.9
Arm thickness (prox. LE in cm)			
Proximal 5 cm		26.6	[20.6–37] ± 2.8
Proximal 10 cm		28.6	[21.7–39.5.5] ± 3.1
Proximal 15 cm		29.9	[21.8–41.7] ± 3.5

**Table 2:** Summary of study measurements

Total (76)	Mean (cm)	[min–max] ± SD	Mean %	[min–max] ± SD
Distance radial N. dorsal (RD) to	in cm		in %	
Acromion	15.1	[12.4–18.1] ± 1.3	49	[45–53] ± 4
AC joint	19.7	[15–25.3] ± 1.9	63	[55–70] ± 6
Lat. epicondyle	15.5	[12.7–17.9] ± 1.3	50	[46–49] ± 4
Med. epicondyle	15.7	[13.5–18.5] ± 1.1	51	[49–51] ± 4
Olecranon fossa	11.5	[9.2–13.7] ± 1.0	37	[33–38] ± 3
Distance radial N. lateral (RL) to	in cm		in %	
Acromion	23.2	[19.2–26.7] ± 1.7	75	[70–74] ± 5
AC joint	26.1	[21.9–30.5] ± 1.9	84	[80–84] ± 6
Lat. epicondyle	6.7	[4.1–8.4] ± 0.8	21	[15–23] ± 3
Med. epicondyle	12.3	[7.8–15.4] ± 1.5	40	[28–42] ± 5
Olecranon fossa	7.6	[5.2–9.9] ± 1.0	25	[19–27] ± 3
Crossing angle between Radial n. and humerus axis	37°	[28–46] ± 4°		

## Discussion

The primary goal of this study was to describe a simple method to identify the radial nerve on the upper arm in the clinical setting. Secondly, we aimed to provide useful information to clinically predict the location of the radial nerve on its most relevant locations on the upper arm (the dorsal and lateral side of the humerus) for settings where time and resources are lacking.

Based on our analysis of 76 measurements, we found that the average distance from the lateral epicondyle to the crossing of the radial nerve on the dorsal side of the humerus (RD) was 15.5 cm ± 1.3, which represented 50% of the humeral length. Similarly, the average distance from the lateral epicondyle to the crossing of the radial nerve on the lateral side of the humerus (RL) was 6.7 cm ± 0.8 cm, corresponding to 21% of the humeral length. Existing studies on the course of the radial nerve in the upper arm have primarily relied on a limited number of cadaver examinations [4–11]. However, due to the lack of standardized reference points for measurement, it is challenging to directly compare our results with those reported in the literature. One study involving 20 cadavers revealed that the radial nerve enters the anterior compartment at approximately the distal 1/3 (33%) of the humerus length [9], while another cadaver-based study reported a 10.9 cm distance between LE and the entry of the nerve in the anterior compartment. In our study, we did not focus on the specific site of nerve entry into the anterior compartment but rather on the precise location of the radial nerve on the lateral side of the humerus. Additionally, slight differences in the reference points used for measurement (lateral acromion vs. posterodorsal corner of the acromion) may account for the disparity in the average distance we report from RL to the lateral epicondyle (21%). Regarding the location of the radial nerve on the dorsal side of the humerus (RD), the only available study for comparison, conducted by Gerwin et al. [6] in 1996, found that the nerve is situated approximately 55% proximal from the lateral epicondyle. This roughly correlates with our findings (50%). The dorsal location of the radial nerve was largely overlooked in other studies. It is important to note that, while cadaver studies provide valuable insights into anatomical structures and their relationships, the findings may not always directly translate to live patients.

### Clinical applications

**Fig. 6:**
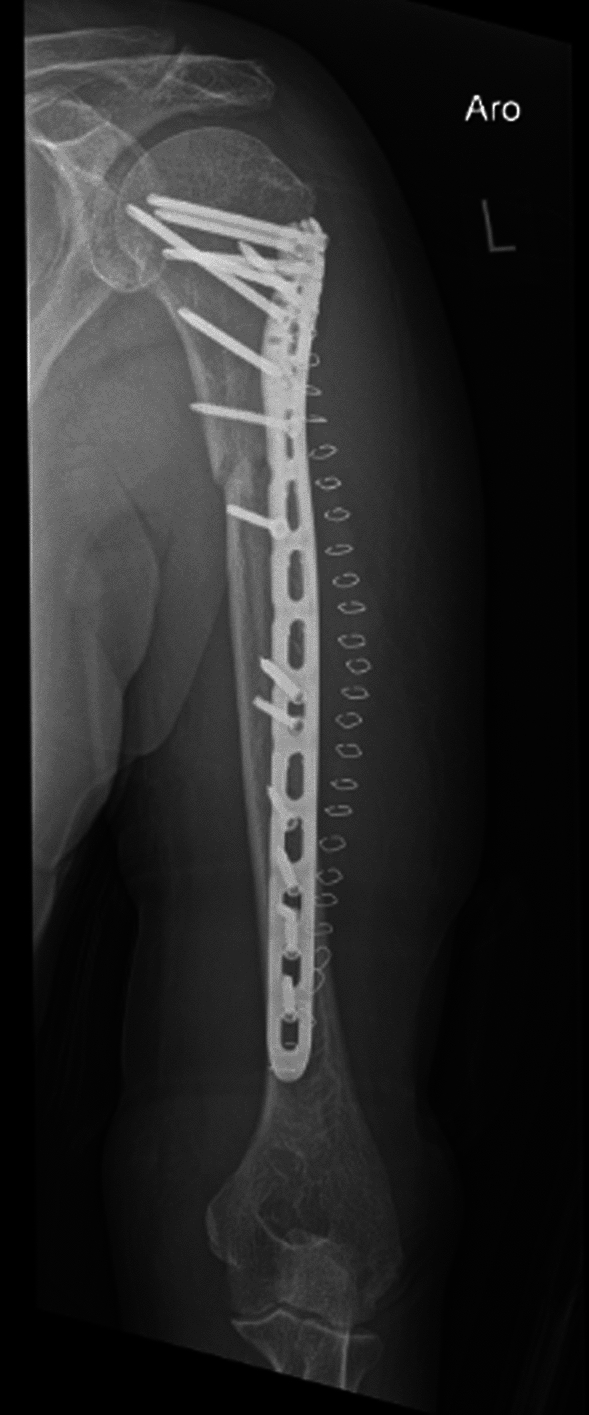
X-ray in external rotation two days after ORIF with a twisted 10 holes-Shaft Long PHILOS^®^ Plate (Synthes, Oberdorf, Switzerland) after proximal humerus shaft fracture [12]. Exact knowledge of the radial nerve’s location before surgery of proximal humerus shaft fractures is essential and can be optimized with ultrasound on the affected or contralateral side

This method has practical implications, particularly in the trauma setting. When performing surgery for proximal and middle shaft fractures of the humerus, where a long straight osteosynthesis plate is commonly used, it is crucial to identify and carefully handle the radial nerve to avoid iatrogenic radial lesions. Even when twisted plates are utilized to avoid the nerve’s course, knowing the nerve’s location on the lateral side of the humerus in advance provides valuable information for plate deformation and nerve avoidance, especially in minimally invasive surgery (Fig. 6).

Similarly, predicting the nerve pathway is beneficial in the dorsal approach for isolated middle shaft fractures of the humerus treated with a dorsal plate, as identifying and protecting the radial nerve is an essential step.

Our results show no difference in the location of the radial nerve on the contralateral side. Therefore, in cases of difficult ultrasound visualization (such as large hematoma or open wounds), indirect measurement could be done on the unaffected arm and then be extrapolated. In the absence of ultrasound, we propose using a 1/2 and 1/5 ratio to estimate the location of the radial nerve on the dorsal and lateral sides of the humerus. By measuring the humerus length from the lateral epicondyle to the dorsolateral corner of the acromion with a measuring tape, fixing the measuring tape on the lateral epicondyle allows us to approximate the position of the radial nerve on the dorsal side of the humerus at 1/2 of the HL (SD ± 1.3 cm), and on the lateral side at 1/5 of the HL (SD ± 0.8 cm). These reference points are easily identifiable even in cases of traumatic swollen arms. This method can also aid in preoperative planning for complex surgeries such as tumor removal.

### Limitations and strengths

Our study methodology has limitations. Measurements were performed by a single examiner, potentially allowing interobserver variability. Though the study population included a broad age, gender and BMI spectrum, it consists of volunteers, which may introduce a selection bias. Factors such as swelling, fractures, tumors, or patients’ inability to cooperate, which can impact measurement accuracy in a clinical setting, were not assessed. However, there are notable strengths. To our knowledge, this is the only study using ultrasound in living individuals presenting the location of the radial nerve on the dorsal and lateral side of the humerus, crucial for trauma surgery. With a sample size of 76 measurements, the study possesses statistically relevant power. The proposed reference points can generally be utilized even in swollen or traumatic scenarios.

## Conclusion

In conclusion, this study aimed to address the need for a standardized protocol to identify and predict the location of the radial nerve in upper arm surgery. The study utilized ultrasound as a cost-effective and easily accessible tool for this purpose.

The measurements revealed that the distance from the lateral epicondyle to the crossing of the radial nerve on the dorsal side of the humerus was, on average, 15.5 cm ± 1.3, corresponding to 50% of the humerus length. Similarly, the distance from the lateral epicondyle to the crossing of the radial nerve on the lateral side of the humerus was, on average, 6.7 cm ± 0.8 cm, corresponding to 21% of the humerus length. The course of the nerve between these two points showed an average angulation of 37° to the humeral axis.

Importantly, gender, body mass index, dominant hand, and arm thickness did not significantly affect the relative location to the radial nerve. Furthermore, the measurements were consistent between the left and right sides within the same individual.

The ultrasound-assisted identification of the radial nerve is easy and fast to perform and can be applied in the clinical context without relevant extra-costs. Preoperative identification of the radial nerve may be particularly useful in orthopedic and trauma surgery of humerus fractures or in tumor resection of the upper arm. By employing the standardized protocol and utilizing the established reference points, surgeons may better identify and protect the radial nerve, reducing the risk of iatrogenic injury.
